# Diagnostic yield of transbronchial cryobiopsy in non-neoplastic lung disease: a retrospective case series

**DOI:** 10.1186/1471-2466-14-171

**Published:** 2014-11-03

**Authors:** Sergej Griff, Nicolas Schönfeld, Wilhelm Ammenwerth, Torsten-Gerriet Blum, Christian Grah, Torsten T Bauer, Wolfram Grüning, Thomas Mairinger, Henrik Wurps

**Affiliations:** Institute of Pathology, HELIOS Klinikum Emil von Behring, Walterhöferstr. 11, 14165 Berlin, Germany; Clinic of Pneumology, Lungenklinik Heckeshorn, HELIOS Klinikum Emil von Behring, Berlin, Germany; Medical Clinic of Pneumology, Gemeinschaftskrankenhaus Havelhöhe, Berlin, Germany; Clinic of Pneumology, HELIOS-Kliniken Schwerin, Schwerin, Germany

**Keywords:** Diffuse parenchymal lung disease (DPLD), Bronchoscopy, Transbronchial biopsy (TBB), Cryotechnique, Histopathology, Usual interstitial pneumonia (UIP)

## Abstract

**Background:**

Due to the small amount of alveolar tissue in transbronchial biopsy (TBB) by forceps, the diagnosis of diffuse, parenchymal lung diseases (DPLD) is inherently problematic, with an overall low yield. The use of cryotechnique in bronchoscopy, including TBB by cryoprobe, has revealed new opportunities in the endoscopical diagnosis of malignant and non-malignant lung diseases.

**Methods:**

To evaluate TBB by cryotechnique for non-neoplastic lung diseases, we analyzed 52 patients (mean age 63 ± 13 years) with unclear DPLD. These individuals underwent bronchoscopy with TBB by cryoprobe. Thereafter histopathological results were compared with the clinically evaluated diagnosis.

**Results:**

No major complications were seen. Mean specimen diameter in the histological biopsies was 6.9 ± 4.4 mm (Range 2 – 22 mm). A correlation between clinical and histopathological diagnoses was found in 79% of cases (41/52). In the case of UIP (usual interstitial pneumonia) pattern, the concordance was 10/15 (66%).

**Conclusion:**

Based on these results TBB by cryotechnique would appear to be a safe and useful method that reveals new perspectives for the endoscopical diagnosis of DPLD.

## Background

Concerning transbronchial lung biopsy (TBB) by forceps, histopathological results depend on specimen size and quality, artificial changes due to the procedure itself, and the amount of alveolar tissue contained in the sample. TBB by forceps typically delivers one or more 1–2 mm sized specimen, often with underrepresentation of alveolar tissue [1–4].

Due to the large variation of indications, as well as different sizes and locations of pulmonary lesions, diagnostic yield of TBB by forceps is severe to describe. Most recent case series specify a diagnostic accuracy of about 50% to 70% [1, 3, 5–11].

Diffuse parenchymal lung diseases (DPLD) are harder to diagnose by TBB with an overall lower yield. Efficacy variations depend on the underlying disease: Sarcoidosis and cryptogenic organizing pneumonia (COP) render fairly good results, whereas usual interstitial pneumonia (UIP), pneumoconiosis, respiratory bronchiolitis associated interstitial lung disease (RB-ILD), non specific interstitial pneumonia (NSIP) and pulmonary histiocytosis X show poor results [1, 4, 10, 12, 13]. This broad range is explained by the diverging importance of alveolar tissue for the histopathological diagnosis.

Due to the small yield of alveoli in TBB by forceps it is not possible to gather further information concerning the histopathological pattern of affected tissue throughout the lung. This problem is well known and has recently been discussed in literature [1, 3, 4, 6, 13–17]. Some papers do show an increase of diagnostic yield depending on the size of the specimen [6, 14], however artificial changes due to TBB by forceps are an important limiting criterion for the pathological diagnosis of DPLD.

Cryobiopsy as a tool in bronchology has been introduced on a routine basis in recent years and has been found to be safe in a routine diagnostic setting [18, 19]. Specimen size has been reported to be larger and diagnostically more valuable due to more alveolar tissue and less artificial changes. In a previous paper morphometrical benefits of cryobioptically obtained lung tissue specimen were shown and perspectives of this method in a daily routine were discussed [20]. There is evidence that cryobiopsies increase efficacy concerning histopathological tumor diagnostics in central malignant lesions [19, 21].

The aim of this study was to evaluate TBB by cryotechnique for non-neoplastic diseases. Our focus was sample adequacy for diagnostic purposes, sample size and proportion of alveolar tissue retrieved, as well as the possibility of histopathological diagnosis of DPLD by cryobiopsy in correlation to clinical diagnoses.

## Methods

This is a retrospective case series (June 2009 – December 2011) of 52 patients with diffuse, interstitial, non-neoplastic lung diseases who underwent flexible, fiberoptic bronchoscopy with transbronchial cryobiopsy. Additionally, all patients had routine diagnostics including lung function evaluation, chest x-ray and thoracic computed tomography (CT-scan).

For the transbronchial cryobiopsy a flexible cryoprobe with a diameter of 1.9 mm was used (flexible Kryosonde diameter 1.9 mm length 900 mm, Erbe Elektromedizin GmbH, Tübingen, Germany), the probe was cooled to a temperature of about – 77°C by carbon dioxide. The flexible bronchoscopy (1 T 160 and 1 T 180, Olympus Corp. Tokyo Japan) was performed under sedation with disoprivan or midazolam and local anaesthesia with lidocaine. The cryoprobe was introduced into the selected area with a distance of approximately 1–2 cm from the thoracic wall under radiological guidance. In this position the cryoprobe was cooled for three to five seconds and then retracted with the attached frozen lung tissue. For each patient one to two specimen were taken and then fixed in 4% buffered formalin.

To avoid incomplete sectioning of specimen particles all biopsies were conventionally processed by serial sectioning of at least 12 H & E stained section steps. Concerning quantity, quality (number of artefacts), and the amount of alveolar tissue, the biopsies were rated by two experienced lung pathologists (SG & TM).

Mirax Viewer Image Software Ver (1, 6) was used for scanning the Hematoxylin-eosine slides by a ZEISS-MIRAX Midi Slide scanning system (Zeiss Microimaging, Oberkochen, Germany and 3DTech, Budapest, Hungary). The total diameter of the biopsy specimens were measured and expressed in μm.

Histopathological changes were rated according to criteria for UIP diagnosis based on the Official ATS/ERS/JRS/ALAT Statement (22). The histological diagnosis of other entities was made by the use of classical criteria for interstial lung diseases (4).

Histopathological results and radiographic images were compared to the patients’ medical history, physical examination and data of pulmonary lung function testing. At last, in an interdisciplinary setting (pathologist, radiologist, pneumologist) a diagnosis was found. Furthermore, complications seen during bronchoscopy were rated.

Statistically, results were expressed as frequencies or as mean ± SD. Chi-square-test was used to compare proportions. The significance level of the analyses was set to 5%, and exact *p* values were reported. Results were expressed using descriptive statistics.

Statistical software (Statistical Package for Social Sciences, Version 14.0; SPSS, Chicago, IL, USA) was used to analyze and process the data on a Windows XP operating system (Microsoft; Redmond, WA, USA).

A waiver for this study was received by the ethics committee of the Charité, Berlin, Germany (“Ethikkommission, Ethikausschuss 1 am Campus Charité – Mitte”) on January 23, 2014.

## Results

Overall, 52 patients with a median age of 63 ± 13 years were analyzed. 36/52 (69%) patients were male, 16/52 (31%) were female. In 41/52 cases (79%) a correlation with clinical and histopathological diagnosis was found. In 11/52 cases (21%) no match could be achieved.

Mean specimen diameter in the histological biopsies was 6.9 ± 4.4 mm (Range 2 – 22 mm). In the specimen, alveolar tissue was found in 48/52 (92%) cases. In 4/52 (8%) cases no alveolar tissue was found. In one of these four cases no histopathological diagnosis could be matched to the clinical diagnosis due to the lack of alveolar tissue. In the other three cases the diagnosis was sarcoidosis, and typical granulomas were found in the bronchial mucosa. The specimens lacking alveolar tissue either contained only bronchial mucosa and sometimes cartilage, or presented themselves as long flat bands of inner bronchial wall lining.

No major complications (pneumothorax, major bleeding >3 minutes) with need of further intervention were reported.

Table 1 shows the list of clinically diagnosed lung diseases, the number of matching histopathological findings and the average diagnostic yield of TBB by forceps reported in literature.

**Table 1:** **Comparison of clinically diagnosed DPLD (with number of cases and matching histopathological findings) and averagely reported diagnostic yield by forceps biopsy**

***Clinical diagnosis***	***Number of cases***	***Matching histopathological findings***	***Average reported diagnostic yield by forceps biopsy***
COP	9	8/9 (89%)	65% (10, 27, 28)
Rheumatoid lung disease	2	2/2 (100%)	
Sarcoidosis	12	10/12 (83%)	69% (10, 28, 29)
Alveolar microlithiasis	1	1/1 (100%)	-
NSIP	1	1/1 (100%)	-
medically-induced lung damages	2	2/2 (100%)	-
HP	7	6/7 (86%)	95% (10)
Pulmonary manifestation of scleroderma	2	1/2 (50%)	-
Histiocytosis	2	1/2 (50%)	-
pANCA-pos. Vasculitis	1	0/1 (0%)	-
IPF	13	9/13 (69%)	34% (1, 10)

The HR-CT images of patients who had the clincal diagnosis of idiopathical lung fibrosis (IPF) or pulmonary manifestation of scleroderma were rated apropos of the radiological criteria for UIP after ATS (American Thoracic Society) and ERS (European Respiratory Society) [22, 23]. Of these fifteen cases, fourteen (93%) showed possible or probable UIP pattern and one (7%) was inconsistent with UIP pattern.

## Discussion

Cryobiopsy in our series proved to be a sufficient tool in the diagnostic processing for various diffuse, parenchymal lung diseases (DPLD). Specifically, the highest diagnostic yields were achieved in patients with sarcoidosis (83%), COP (89%), and hypersensitivity pneumonia (HP, 86%). Comparable results with the use of transbronchial forceps biopsy have been reported in recent years [1, 3, 5–10] (see Table 1). These good results are probably due to the location of granulomatous or other characteristic changes close to or within the bronchial wall.

Nevertheless clear distinctions became obvious between diseases that required the recognition of a gross histological pattern (UIP, NSIP, RB-ILD) and all others.

It has generally been assumed that transbronchial lung biopsies cannot be used for the diagnosis of UIP [10]. A hallmark characteristic of UIP is the patchy involvement of lung tissue, so that areas of involved parenchyma and unaffected alveoli stand next to each other. Furthermore, UIP is characterized histologically by fibrosis and chronic inflammation, i.e. features that are usual unspecific findings located in the peribronchial tissue.

In a study by Berbescu et al. [1], 22 patients with UIP pattern assessed by open lung biopsy were retrospectively analyzed concerning a pre-op achieved TBB. This revealed a characteristic histopathological UIP pattern in nine cases. Berbescu et al. concluded [1] that certain characteristic features of UIP, such as the patchwork pattern of involvement by fibrosis and temporal variability with fibroblast foci, collagen, and honeycomb changes, previously thought to be recognizable only on surgical lung biopsy specimen, can sometimes be seen on TBB specimen. The patchwork pattern is typically characterized by normal alveoli in close relation to areas of interstitial fibrosis. Its presence helps to distinguish the changes to nonspecific peribronchial fibrosis where there is a gradual transition from normal to abnormal.

In our series of transbronchial cryobiopsies an UIP pattern was diagnosed in two-thirds (10/15, 67%) of the cases (IPF, pulmonary manifestation of scleroderma, see Table 1). This improves the diagnostic yield of TBB for UIP pattern in comparison to data described in literature by up to 50% (see Table 1).

**Figure 1:**
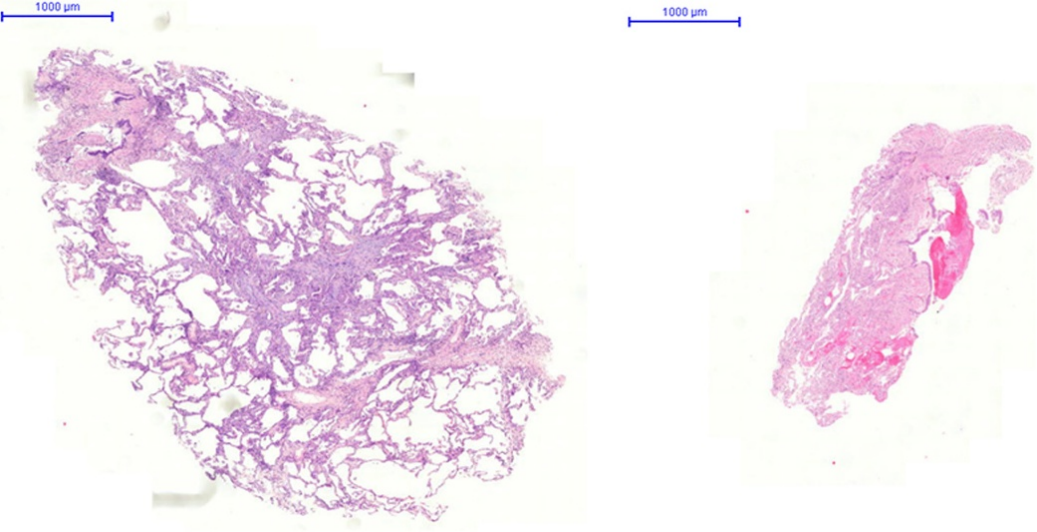
**Comparison of TBB by cryoprobe (left) and forceps (right): significant differences in size and quality.**

**Figure 2:**
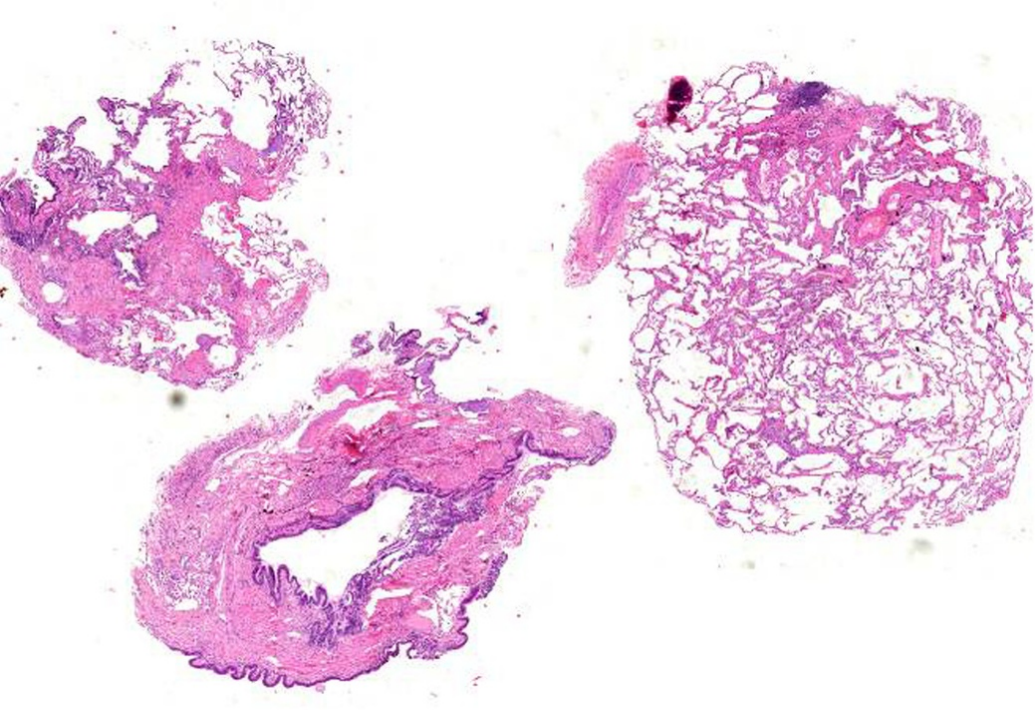
**Patient with radiological UIP pattern.** Overview of transbronchial cryobiopsy: patchy involvement of fibrosing process next to unaffected lung tissue.

**Figure 3:**
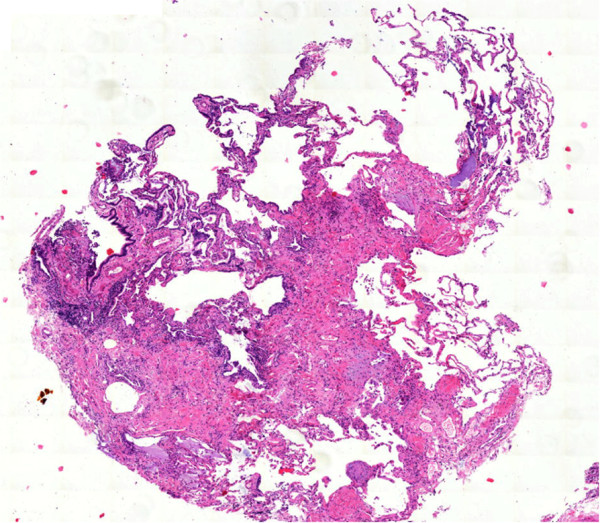
**Transbronchial cryobiopsy: architectural distortion of lung tissue with scaring next to normal lung parenchyma.**

**Figure 4:**
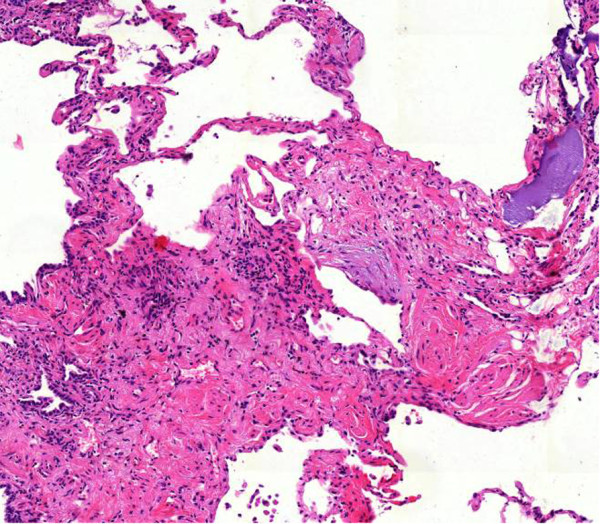
**Transbronchial cryobiopsy: active ongoing fibrosis (fibroblast focus) as an expression of “temporary variegation”.**

Cryobiopsy specimen tend to be even larger than transbronchial forceps biopsy specimen, and contain more and larger amounts of alveolar tissue [21]. In a previous study the number of alveolar spaces necessary for an adequate biopsy was defined as 20 [6]. This criterion is likely to be fulfilled in most of the cryobiopsy specimen ([21]; unpublished data). However, transbronchial cryobiopsy, as well as TBB by forceps, fail to deliver the diagnosis of UIP in a significant proportion of patients. This may be due to the distance seen frequently between the bronchial wall and typical histological changes, such as fibroblast foci, which are located deeply in the alveolar parenchyma [1] (Figures 1, 2, 3, 4).

Despite the encouraging findings about cryobiopsy these results do not yet command a recommendation of transbronchial cryobiopsy as a standard procedure in the processing of suspected pulmonary fibrosis. Nevertheless, the current problem of distinguishing between HP with UIP pattern and IPF (which radiologically speaking cannot be securely discriminated [24]) could be solved by the use of cryobiopsy with greater specimen size. Furthermore, there is a greater chance for detection of granulomas or other characteristic histopathological features [25–29]. Therefore larger prospective and comparative series must be evaluated before a general clinical algorithm can be proposed. Meanwhile, in those individual patients where cryobiopsy has revealed the full pattern of UIP, open lung biopsy is unnecessary if histology and clinical data engender a clear diagnosis.

## Conclusions

Cryobiopsy could improve the results reported on conventional transbronchial forceps biopsy. Nevertheless, previously reported series are small and prospective comparisons do not exist. Such studies could even reveal that eventually less cryobiopsy pieces per patient are necessary as compared to transbronchial forceps biopsies. For the latter most of the authors recommend four biopsies per bronchoscopy during the processing of diffuse lung disease. In our present series only 1–2 cryobiopsy specimen were sampled as a rule.

The high diagnostic yield and the lack of any major complication in our series encourages one to proceed with larger studies and to establish transbronchial cryobiopsy within routine clinical algorithms in the diagnostic of diffuse, parenchymal lung disease.

## Abbreviations

ATS: American Thoracic Society
COP: Cryptogenic organizing pneumonia
CT: Computed tomography
DPLD: Diffuse, parenchymal lung disease
ERS: European Respiratory Society
HP: Hypersensitivity pneumonia
HR-CT: High resolution computed tomography
IPF: Idiopathic pulmonary fibrosis
NSIP: Non specific interstitial pneumonia
pANCA: Perinuclear anti-neutrophil cytoplasmic antibodies
RB-ILD: Respiratory bronchiolitis associated interstitial lung disease
TBB: Transbronchial biopsy
UIP: Usual interstitial pneumonia.
